# Long-Term Performance of Ultrafiltration Membranes: Corrosion Fouling Aspect

**DOI:** 10.3390/ma16041673

**Published:** 2023-02-16

**Authors:** Wirginia Tomczak, Marek Gryta

**Affiliations:** 1 Faculty of Chemical Technology and Engineering, Bydgoszcz University of Science and Technology, 3 Seminaryjna Street, 85-326 Bydgoszcz, Poland; 2 Faculty of Chemical Technology and Engineering, West Pomeranian University of Technology in Szczecin, ul. Pułaskiego 10, 70-322 Szczecin, Poland

**Keywords:** corrosion, fouling, membrane cleaning, oil-in-water emulsion, polymeric membranes, ultrafiltration

## Abstract

The past decade has seen a rise in the importance of the ultrafiltration (UF) technique in the separation of various complex solutions. However, the fouling phenomenon is the main limitation to faster process development. To the best of the authors’ knowledge, the present paper is the first to aim to identify the role of corrosion fouling in long-term UF. For this purpose, polyvinylidene fluoride (PVDF) and polyethersulfone (PES) membranes were used. The investigations were carried out with the use of both pilot-scale and laboratory-scale units. Results obtained in the present study have clearly demonstrated that the oil concentration has a significant impact on the process performance. Indeed, it has been noted that a reduction in oil concentration from 160 to 100 mg/L resulted in an increase in the PVDF membrane flux from 57 to 77 L/m^2^h. In addition, it has been shown that the feed temperature has a significant influence on the UF performance. Importantly, it has been shown that corrosion fouling is of vital importance in UF membranes. Indeed, corrosion products such as iron oxides contaminated the membrane surface leading to an irreversible decrease in the UF process performance. In addition, it has been found that repeating the chemical cleaning of the membrane units significantly reduced the intensity of the fouling phenomenon. However, the complete elimination of its effects was not achieved. Therefore, it has been indicated that cleaning agents recommended by membrane manufacturers do not remove corrosion products deposited on the membrane surface. Undoubtedly, the obtained results can be used in the design of UF units leading to the extension of membrane installation lifetime.

## 1. Introduction

Oil-in-water emulsions are a huge waste stream produced by the petrochemical, metallurgical, food, pharmaceutical, and cosmetics industries [1,2,3]. Hence, the separation of oily emulsions is crucial from both environmental and economic aspects [4]. This process depends on several factors, such as the content and physical nature of oil as well as the chemical nature of other components [5]. It is well known that the separation of oily emulsions can be successfully performed by membrane processes. Indeed, the ultrafiltration (UF) technique was demonstrated to be an efficient and effective method for separating oil-in-water emulsions with the use of both ceramic and polymeric membranes [6,7,8,9,10,11,12,13].

UF is a low-pressure-driven (0.3–1 MPa) membrane process in which the separation of the dissolved matter is based on a size exclusion/sieving mechanism. It has become increasingly popular due to its several advantages such as: high oil rejection, low energy requirement and cost, facile operation, and ease of scale-up [6,14,15,16,17]. However, the separation performance during the filtration of oily emulsions is severely limited by the fouling phenomenon. This is mainly caused by the accumulation and adsorption of oil and particles on the membrane surface as well as membrane pore blocking [5,15]. Moreover, fouling leads to a reduction in membrane lifetime and permeate quality as well as an increase in operation costs [18]. It has been widely documented that fouling is influenced by several factors, for instance, feed–membrane surface interaction, membrane pore size and surface charge and oil content, as well as process conditions [19,20].

Undoubtedly, the inevitable consequence of fouling is a decline in process performance in terms of membrane hydraulic permeability. For this reason, nowadays, considerable efforts are being undertaken to find methods to prevent or reduce this phenomenon. The choice of the most suitable procedure for membrane cleaning depends mainly on the foulant type, membrane material, and module configuration [21]. Frequent chemical cleaning is the most common method of membrane regeneration; however, as has been indicated by Faibish and Cohen [22], the cleaning of irreversibly contaminated membranes requires harsh conditions and, often, the recovery of the initial membrane performance cannot be achieved. Moreover, it should be pointed out that aggressive aqueous environments may lead to the corrosion of the stainless steel elements of membrane systems, which, consequently, may result in equipment failure and/or deposition of corrosion products on the membrane surface. The importance of this point is that corrosion fouling may lead to both membrane clogging and damage to its surface [23]. Publications focused on membrane fouling mainly demonstrate the impact of the feed composition and membrane properties on the flux decline, while, the influence of the entire membrane installation structure on the membrane process performance has not been yet fully evaluated.

It has to be highlighted that the studies focused on the significance of corrosion products in membrane processes have been mainly limited to reverse osmosis (RO) [24,25,26] and membrane distillation (MD) systems [27,28]. For instance, Xu et al. [24] analyzed the corrosion of stainless steel valves in a RO system. It has been shown that the corrosion products were composed of several compounds, such as iron oxides, iron hydroxides, green rust, chromium oxide, calcium carbon oxide, and siderite. Moreover, it has been determined that the corrosion included pitting, cracking, and accumulation of products on the alloy surface. Ling et al. [25] investigated the catalytic effect of iron oxides on the degradation of polyamide (PA) reverse osmosis membranes. Their results demonstrated that the degradation of the membrane used was accelerated by both increasing the concentration of hydrogen peroxide (H_2_O_2_) and the addition of chloride ions that promote the corrosion of system components and, consequently, led to the deposition of iron oxides on the membrane surface. In turn, in [28] it has been shown that during the long-term MD process of saline and acid solutions, steel elements of an installation underwent corrosion leading to the membranes fouling.

It should be pointed out that, although the UF process has been investigated intensively for oil-in-water emulsion separation, the issue of the fouling phenomenon caused by the corrosion products is still poorly studied. Hence, in order to improve the UF process performance, a requirement to detect iron that may lead to the formation of cake deposits on the membrane surface is postulated. In view of what it reveals, to the best of the authors’ knowledge, the present paper is the first to aim at identifying the role of corrosion fouling in the long-term UF process. For this purpose, both pilot-scale and laboratory-scale units were used.

## 2. Materials and Methods

### 2.1. Ultrafiltration Membranes

The separation of oil emulsions has been conducted with the use of two types of polymeric membranes, the characteristics of which are given in Table 1. The PCI B1 tubular module was equipped with 18 membrane tubes FP100 (diameter, length, and total membrane area of 1.25/1.27, 1.2 m, and 0.85 m^2^, respectively). The membranes were made from polyvinylidene fluoride (PVDF) and manufactured by PCI Membranes (Kostrzyń, Poland). The investigations were also carried out with the use of flat UE50 membranes made from polyethersulfone (PES) manufactured by TriSep Corporation (CA, SA).

### 2.2. Ultrafiltration Units

#### 2.2.1. Pilot-Scale Unit

**Figure 1:**
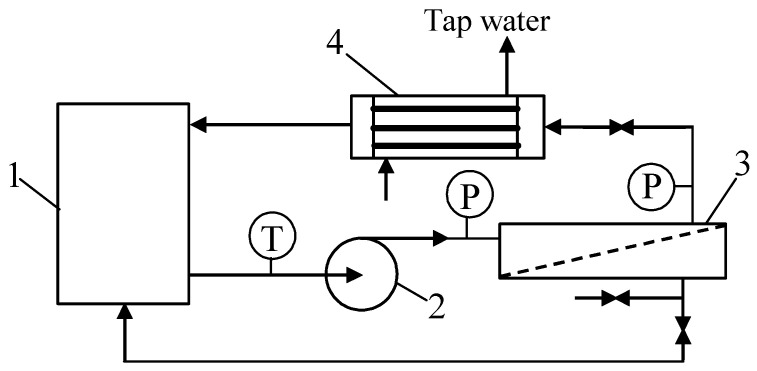
Experimental set-up of the pilot-scale UF unit with the tubular membrane module. 1—feed tank, 2—pump, 3—UF module, 4—heat exchanger, T—thermometer, P—manometer.

The PCI B1 tubular module equipped with the FP100 membranes (Table 1) was mounted in the pilot-scale installation shown in Figure 1. The UF studies were performed at transmembrane pressures (TMPs) in the range of 0.1 to 0.4 MPa. The feed was pressurized by a centrifugal pump (type VNR-8, Grundfos, Denmark) and flowed inside the tubular membranes with a velocity of 1.2 m/s. The feed temperature was between 298 and 308 K. Temperature control was ensured by a shell-and-tube cooler made of 1H18N9T stainless steel (designed also as AISI 321) through which the feed flowed. The remaining elements of the installation were made of polyvinyl chloride (PVC).

#### 2.2.2. Laboratory-Scale Unit

**Figure 2:**
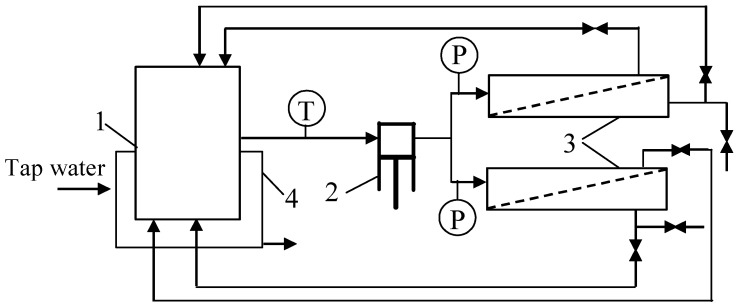
Experimental set-up of the laboratory-scale UF unit with two plate membrane modules. 1—feed tank, 2—pump, 3—module, 4—water cooling, T—thermometer, P—manometer.

The UF process was also carried out in a laboratory-scale installation (Figure 2) in which two flat modules made of acid-resistant steel (AISI 316) with UE50 membranes (Table 1) were installed. The installation was equipped with a 3CP Stainless Steel Plunger Pump model 3CP1221 (CAT PUMPS, Hampshire, UK). The channels in the modules (dimensions 40 × 60 × 0.7 mm) were filled with polypropylene (PP) net-spacer (orthogonal, 20 mesh). The other elements of the installation were made of glass and polypropylene tubes. The UF studies were performed under closely controlled experimental conditions. The TMP was in the range of 0.05 to 0.3 MPa. The feed flow and temperature were 0.63 m/s and 298 K, respectively.

### 2.3. Feed Solutions

Tap water (580 µS/cm) purified using the nanofiltration (NF) process was used to prepare the feed. The concentrations of major ions in the tap water and obtained NF permeate (48 µS/cm) are shown in Table 2.

Oil-in-water emulsion was prepared by adding oil emulsion concentrate to the water in the feed tank (100 L). For this purpose, the oil collected from the surface of the bilge water in a ship engine room was mixed with deionized water (Elix 3-Millipore, Burlington, MA, USA) using ultrasonics. The method of emulsion preparation was described in detail in a previous work [29]. In the present study, the experiments were carried out for the initial oil concentration in water in the range of 50 to 200 mg/L.

### 2.4. Analytical Methods

The permeate flux [L/m^2^h] was determined according to the following equation:

(1) $$J=\frac{V_{P}}{S\cdot t}$$

where V_P_ [L] is the volume of permeate, S is the active surface of the membrane [m^2^], and t [h] is the unit of time.

The oil rejection efficiency [%] was determined as follows:

(2) $$R=\left(1-\frac{C_{P}}{C_{f}}\right)\cdot 100\%$$

where C_p_ [mg/L] and C_f_ [mg/L] are the measured oil concentrations of the permeate and feed. respectively.

Samples collected from the feed, concentrate, and permeate were analyzed in terms of oil concentration, electrical conductivity, and composition. The oil concentration was determined based on the infrared (IR) analysis method using an OCMA 310 apparatus (Horiba, Kyoto, Japan). The electrical conductivity of solutions was measured with a 6P Ultrameter (Myron L Company, Carlsbad, CA, USA). Solution composition was measured using an 850 Professional IC ion chromatograph (Herisau Metrohm AG, Herisau, Switzerland). The membrane morphology and composition of deposits were investigated using a SU8020 (Hitachi High Technologies Co., Tokyo, Japan) scanning electron microscope (SEM) coupled with energy dispersion spectrometry (EDS). Details of the above-mentioned methods have been presented in detail in previously published papers [12,29].

## 3. Results and Discussion

### 3.1. Pilot-Scale Unit

The pilot-scale unit (Figure 1) equipped with the FP100 membranes (Table 1) was used to investigate the impact of transmembrane pressure (TMP), feed temperature, and flow rate, as well as oil concentration, on both the permeate flux and oil retention degree. For this purpose, the installation was operated periodically over about 5 years. The obtained results have been presented in a previous paper [30].

**Figure 3:**
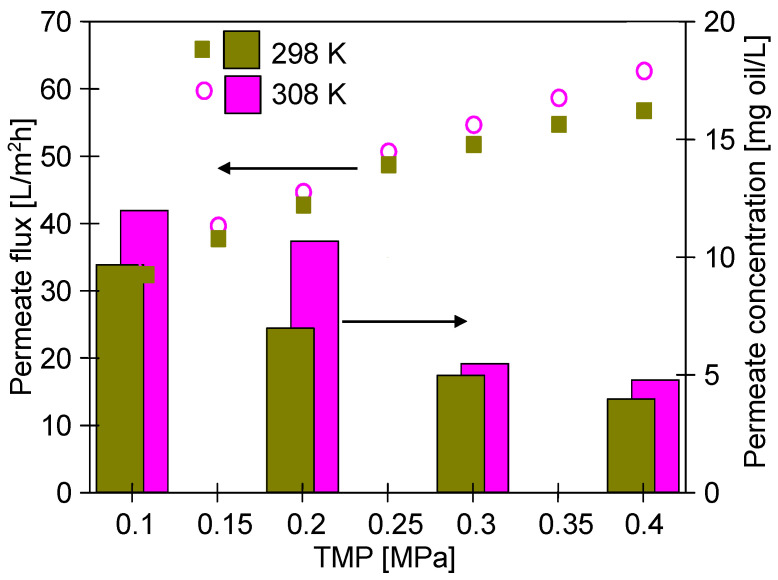
The impact of TMP and feed temperature on the permeate flux and oil concentration in the permeate. Feed concentration 160 mg oil/L. Tubular membrane FP100.

Exemplary results obtained during the UF process of a feed containing 160 mg/L of oil are presented in Figure 3. In the UF process, the permeate flux is determined according to Darcy’s law:

(3) $$J=\frac{\mathrm{TMP}}{\mu (R_{m}+R_{f})}$$

where µ [Pa·s] is the permeate viscosity and R_m_ [m^−1^] and R_f_ [m^−1^] are the intrinsic membrane resistance and resistance due to fouling, respectively.

It has been determined that increasing the TMP led to an increase in the obtained permeate flux (Figure 3). As expected, the noted increase was not linear, indicating an additional resistance R_f_ in addition to the membrane resistance R_m_. This can be explained by the fact that the compounds present in the feed formed a deposit layer on the membrane surface, which consequently results in an increase in the total resistance value. In turn, with regards to the impact of temperature on the process performance, it should be noted that, generally, the viscosity of the solution decreases with the temperature [31,32], hence, according to Darcy’s law (Equation (3)) increasing the feed temperature T_F_ from 298 to 308 K resulted in an increase in the permeate flux. Importantly, this result substantiates previous findings in the literature [33,34,35,36] wherein it has been shown that during the UF of oil-in-water emulsion, the temperature has a significant influence on the permeate flux. Worthy of note, the reduced oil viscosity facilitated its penetration through the membranes pore and, as a result, its retention degree at 308 K slightly decreased. Nevertheless, it was still high and exceeded 93%.

**Figure 4:**
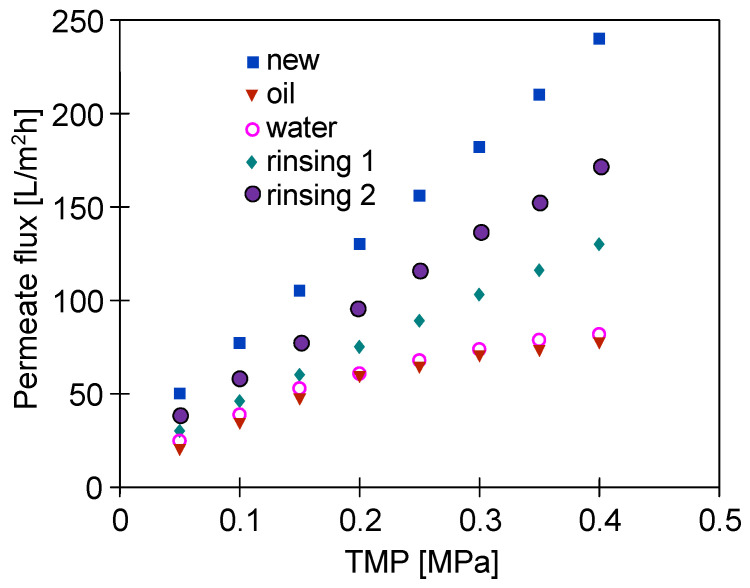
The impact of TMP on the permeate flux. Feed: new–distilled water, oil-NF permeate + 100 mg oil/L, water-distilled water, rinsing-NF permeate. New–virgin FP100 membrane, rinsing 1 and rinsing 2-after membrane cleaning with the P3 Ultrasil 11 solution. The first year of the module exploitation.

Moreover, results obtained in the present study have clearly shown that the oil concentration has a significant impact on membrane performance. Comparing the results shown in Figure 3 and Figure 4 (TMP = 0.4 MPa, T_F_ = 308 K) it can be clearly seen that the reduction in oil concentration from 160 to 100 mg/L resulted in an increase in the flux from 57 to 77 L/m^2^h. According to [37,38], this finding can be explained by the fact that the feed containing oil of a higher concentration leads to an increase in the total resistance due to the formation of a thicker oil layer on the membrane surface.

It has been found that rinsing the membrane module with water only slightly increased the module performance, which indicates strong adsorption of oil contaminants on the membrane surface (Figure 4). Conversely, a significant increase in the module performance was obtained after chemical cleaning with P3 Ultrasil 11 solution agent (pH = 10.8). This solution contained sodium hydroxide, EDTA, and anionic and non-ionic surfactants. However, the recorded values of permeate flux under all applied TMP (Figure 4, ‘rinsing’) were lower than those observed for the new membrane (Figure 4, ’new’). For instance, the performed membrane cleaning allowed the flux to increase to 130 L/m^2^h under a TMP of 0.4 MPa, while the initial flux was equal to 240 L/m^2^h. Worthy of note, repeating the membrane cleaning operation increased the maximum flux to 170 L/m^2^h. Based on this finding, it can be concluded that, after the filtration of real solutions, the maximum performance of the membranes irretrievably stabilized at a lower level than that noted for the new membranes. Based on the literature review, it should be noted that the P3 Ultrasil 11 solution has been widely used as a chemical cleaning agent for UF membranes fouled with components of various feed solutions, such as oily wastewaters [12,30], separated digestate [39], orange juice [40,41], *Fucus vesiculosus* extracts [42] and spent sulfite liquor [43].

**Figure 5:**
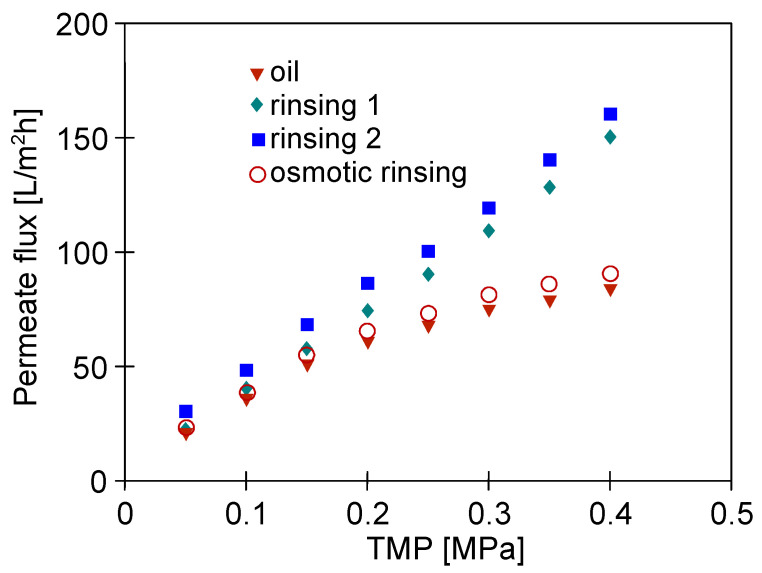
The impact of TMP on the permeate flux. Feed: oil-NF permeate + 100 mg oil/L, NF permeate, rinsing 1 and rinsing 2. Rinsing 1, 2-after the membrane cleaning with the P3 Ultrasil 11 solution. Osmotic rinsing-membrane soaked in NF permeate for 12 h. The 5th year of the module exploitation.

Figure 5 shows the results obtained during the 5th year of the module operation. Importantly, it can be indicated that during the separation of the feed containing 100 mg/L of oil (Figure 5, ‘oil’), the changes in the permeate flux as a function of TMP were similar to that obtained four years earlier (Figure 4, ‘oil’). It has been found that soaking the module in the NF permeate for 12 h (osmotic rinsing) allowed a slightly greater increase in the flux to be obtained than that noted after membrane rinsing with the NF permeate for 60 min. After the first run of membrane chemical cleaning with P3 Ultrasil 11 solution, the maximum flux at a TMP of 0.4 MPa was equal to 150 L/m^2^h and increased to 160 L/m^2^h when the chemical cleaning was repeated (Figure 5, rinsing 2). Thus, these values were close to the maximum value (170 L/m^2^h) obtained after membrane chemical cleaning in the first year of the module operation (Figure 4). These results prove the high stability of the FP100 membranes, which indicates that they can be successfully used for the separation of oily emulsions [30].

**Figure 6:**
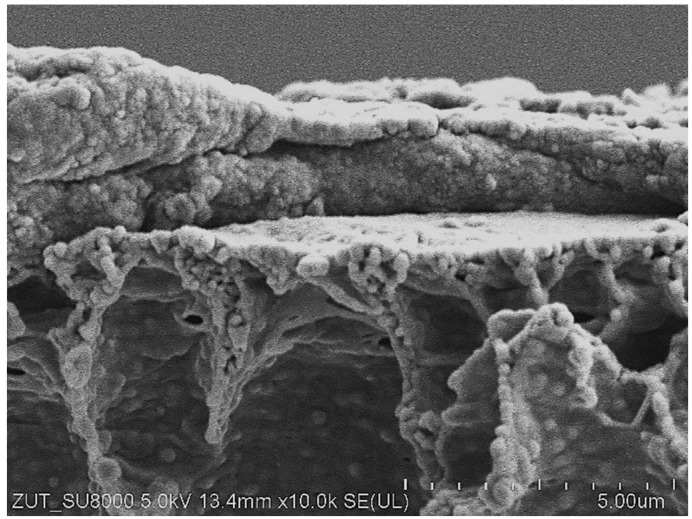
SEM image of the FP100 membrane (a cross-section) covered with sediment. Membrane sample collected after 5 years of the UF module exploitation.

**Figure 7:**
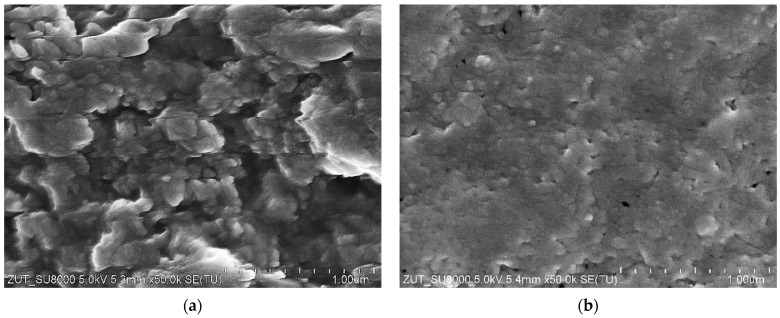
SEM images of the fouled FP100 membrane surface collected from the module after 5 years of operation: (**a**) Covered by the deposit; (**b**) After washing with the use of 35% HCl solution.

As demonstrated above (Figure 4 and Figure 5), the maximum performance of the new FP100 membranes irreversibly decreased during the conducted investigations. SEM studies of the membrane samples showed that this can be attributed to the formation of a 2–5 µm thick deposit on the membrane surface (Figure 6). The cross-section of the sediment was multi-layered, which indicates that the deposit was formed in several stages. The analysis performed under high magnification (50 k) showed that the deposit was porous (Figure 7a), hence, despite its considerable thickness, water could still penetrate the membrane.

**Figure 8:**
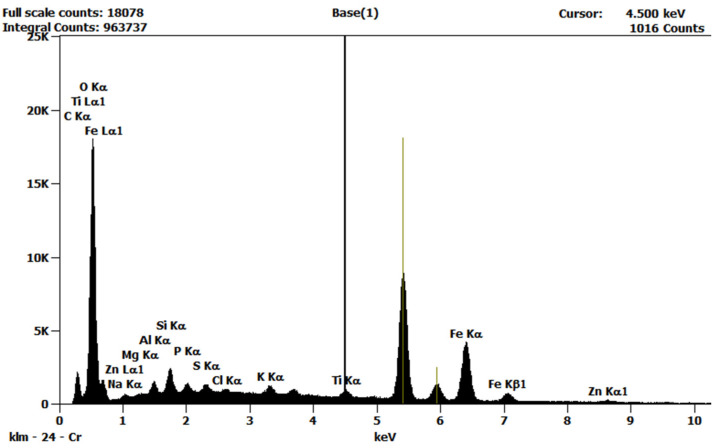
SEM-EDX spectrum. FP100 membrane.

The deposit composition determined by the SEM-EDS method is presented in Table 3 and the EDX spectrum in Figure 8. Surprisingly, a significant amount of Fe was noted. Based on the large amount of O detected, it can be concluded that Fe occurred in the deposit in the form of oxides. Generally, the corrosion process of steel is a complex reaction including both anodic metal oxidation and cathodic oxygen reduction [44]. Consequently, as has been indicated in [23], oxidized metals may occur in the following forms: Fe_2_O_3_, FeO, or Fe_3_O_4_. It can be assumed that Fe came from the corrosion of the heat exchanger, which is the only element of the installation made of steel (apart from the pump).

The pilot-scale unit was used periodically. Indeed, the tests were usually carried out at intervals of 2–3 days and sometimes longer than 30 days. Before each downtime, in order to protect the membranes, the installation was rinsed with the 0.25% sodium disulfite (Na_2_S_2_O_5_) solution for 30 min. Then, the solution was removed from the installation; however, its remains wet the installation walls. Subsequent studies started with rinsing the installation with the NF permeate. It was observed that the color of the feed became brown, which could indicate that corrosion products were washed out from the heat exchanger surface. Since the permeate valve was closed during rinsing, it can be concluded that the contact of the contaminated feed with the membrane surface led to the deposition of iron-containing impurities on it.

As indicated above, the surface of the contaminated membranes was brown, indicating the presence of insoluble iron oxides [45]. During analysis, the deposit was dissolved by immersing the membrane sample in a 35% hydrochloric acid (HCl) solution. As a result, the solution turned green, which is characteristic of ferric chloride. The SEM images of the FP100 membrane are shown in Figure 7. The studies confirmed the effectiveness of the applied cleaning method (Figure 7b). However, it is obvious that the use of a concentrated acid solution would require a special chemically resistant construction of the UF installation, which from a practical point of view, eliminates the presented methodology.

### 3.2. Laboratory-Scale Unit

The results obtained during the long-term exploration of the UF pilot-scale installation indicate that not only the feed components but also the corrosion products of the installation components may cause fouling. Particularly, the process of membrane cleaning can contribute to this phenomenon. Indeed, the proposed methods in the literature for cleaning the modules are often carried out at high temperatures or with the use of acids, such as nitric acid (HNO_3_) [5,46,47,48]. For this reason, with regard to metal elements, it is necessary to make them of corrosion-resistant steels, such as acid-resistant steels. It is worth emphasizing that, although they do not completely eliminate the corrosion process, they ensure that it proceeds much more slowly than with structural steel. Hence, it should be pointed out that more research focused on the materials and their effect on corrosion in UF plants is required.

To investigate the impact of steel corrosion on the fouling phenomenon, the laboratory-scale unit (Figure 2) with flat modules made of AISI316 stainless steel and a 3CP1221 acid-resistant piston pump was used. Before installing the membranes, the installation was chemically cleaned and rinsed with the NF permeate for 3 days. The solution was replaced at least twice per day. The first chemical cleaning run was carried out with the use of 0.3% P3 Ultrasil 11 solution for 1 h. The second run was performed the next day with a 2% citric acid solution for 1 h. The residues of the chemical agents were removed from the installation by flushing with NF permeate (5 L).

**Figure 9:**
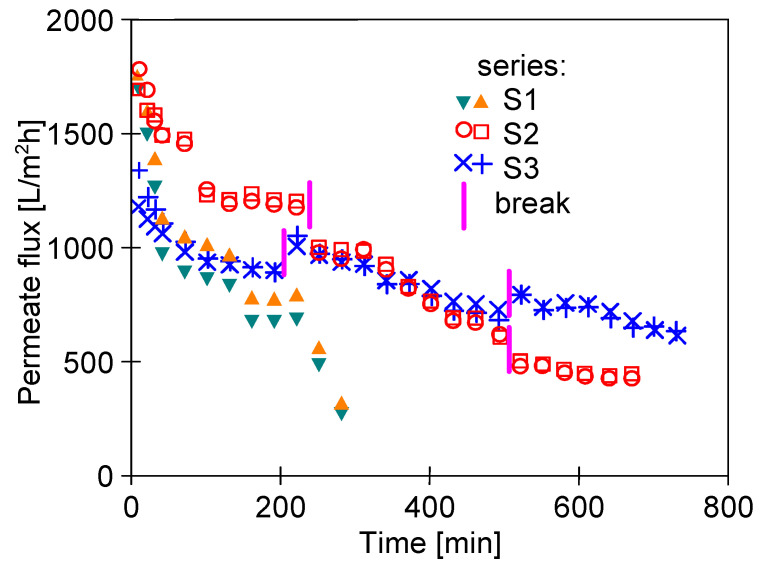
Permeate flux changes during the membrane module operation. Feed: permeate NF. TMP = 0.3 MPa, T = 298 K, feed flow = 0.5 m/s. Break-osmotic rinsing.

After flushing the installation, the UE50 membranes were installed in the modules. In both modules, the channel surface was clean (silvery) with no traces of corrosion products. NF permeate was used as the feed and the UF process was carried out for 5 h under a TMP of 0.3 MPa. The obtained results are shown in Figure 9 (series S1). During the first 60 min of the process, a rapid decline in the permeate flux was observed. Indeed, it has been noted that the permeate flux decreased from 1700 to 1000 L/m^2^h. In the following hours, after the stabilization of module performance, the flux decreased rapidly again, which was caused by the fouling phenomenon.

**Figure 10:**
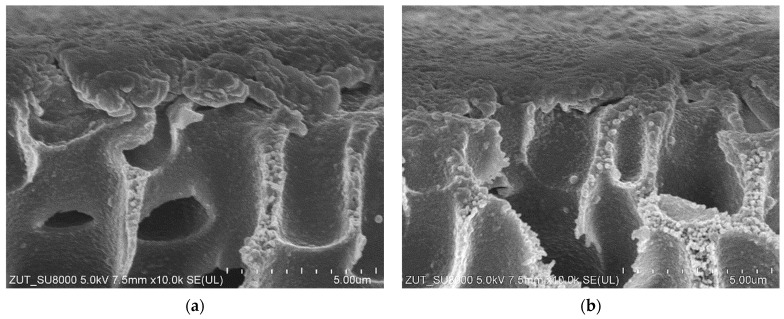
SEM image of the UE50 membrane cross-section and membrane surface (at the top of the photo) from the feed side: (**a**) Membrane sample after the series S1 (Figure 9); (**b**) Sample of the membrane cleaned after the series S2 (Figure 9).

**Figure 11:**
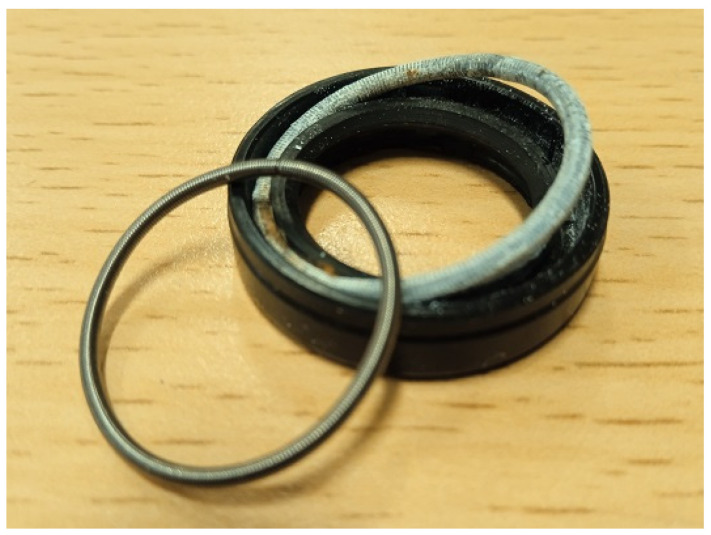
Photo of the springs inside the rubber seals. Left side: new spring, right side: spring used.

It was observed that after the end of the S1 run, the membrane surface on the feed side changed from white to slightly brown. Moreover, SEM studies confirmed that a thin layer of deposit had formed on the membrane surface (Figure 10a). Importantly, the SEM-EDS analysis (Table 4) showed the presence of Fe in the deposit. Due to the short period of the process operation, its content was much less than that recorded in the case of FP100 membranes (Table 3). After the process run, the channels in the membrane modules and the feed tank were clean, without traces of corrosion products. However, after dismantling the pump, corrosion of the springs inside the rubber seals (Figure 11) mounted on the ceramic plungers was found. The surfaces of all internal pump components were thoroughly cleaned and reassembled with new O-rings and seals.

**Figure 12:**
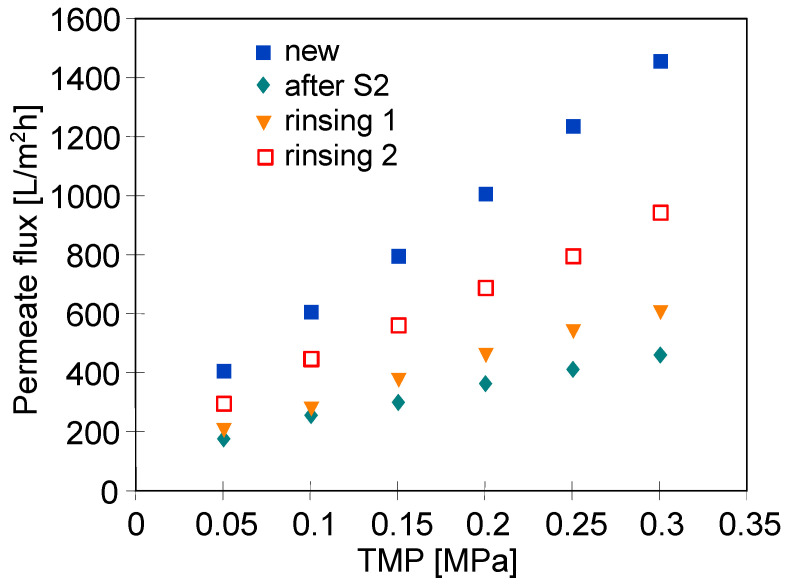
Cleaning efficiency of the UE50 membranes fouled during measurement series S2 (Figure 9). P3 Ultrasil 11 (rinsing 1), 5% citric acid (rinsing 2).

After installing the new UE50 membranes, a much slower decrease in permeate flux was noted during filtration (Figure 9, series S2). Therefore, it can be indicated that the main reason for the fouling during the S1 series was the corrosion of the springs mounted inside the seals. After 5 h of the process run, the flux stabilized at the level of 1200 L/m^2^h. After completing the measurement series, the installation was rinsed with a new portion of NF permeate, which remained in the installation overnight (12 h). After restarting the process, the flux stabilized at a lower level (1050 L/m^2^h) and after 2 h it decreased to 600 L/m^2^h. Following a night break (12 h), the permeate flux stabilized at 450 L/m^2^h. Then, the membranes were cleaned with P3 Ultrasil 11 solution for 30 min, which slightly improved the process performance (Figure 12, rinsing 1). A second chemical cleaning was performed using the 5% citric acid solution, resulting in a further increase in the flux (Figure 12, rinsing 2). However, in each case, the initial performance was not restored.

After removing the membranes from the modules, their surface color was only a light shade of brown, demonstrating a significant reduction in the fouling phenomenon. However, it indicated the presence of corrosion products. In order to clean the installation again, it was rinsed with a citric acid (C₆H₈O₇) solution and subsequently, with a sodium phosphate (Na_3_PO_4_) solution, after which the installations were thoroughly rinsed with NF permeate.

The results obtained with the use of the third set of UE50 membranes (Figure 9, series S3) showed a more stable process performance. After three days of the studies, the flux stabilized at the level of 700 L/m^2^h. After each night’s (12 h) break, permeate flux increased and then, decreased again. Such a process course could have resulted from the membranes compression, which expanded during the overnight stop. It is worth mentioning that SEM studies of the membrane samples after the measurement series confirmed that there were fewer deposits on the membrane surface than observed in the previous series (Figure 10). This effect was obtained after repeated cleaning and rinsing of the installation. The obtained results demonstrate that even trace amounts of impurities (especially corrosion products) can have a significant impact on the results.

## 4. Conclusions

The present study aimed at identifying the role of corrosion fouling in the long-term UF process. For this purpose, PVDF and PES membranes were used. The experimental investigations were carried out with the use of both pilot-scale and laboratory-scale units. It has been clearly demonstrated that the oil concentration has a significant impact on the process performance. The reduction in oil concentration from 160 to 100 mg/L resulted in an increase in the PVDF membrane flux from 57 to 77 L/m^2^h. In addition, it has been shown that the feed temperature has a significant influence on the UF performance. Importantly, it has been found that corrosion fouling is of vital importance in the UF process. Indeed, corrosion products were one of the deposit components formed on the UF membranes surface causing irreversible fouling and, consequently, a significant decrease in the process performance. Moreover, it has been found that repeating the chemical cleaning of the membrane units significantly reduced the intensity of the fouling phenomenon; however, complete elimination of its effects was not achieved. Therefore, it can be indicated that even trace amounts of corrosion products have an impact on changes in the performance of UF modules. Finally, it can be expected that the cleaning agents recommended by membrane manufacturers, such as P3 Ultrasil 11, do not remove corrosion products deposited on the membrane surface, such as iron oxides. The obtained results can be used in the design of UF units leading to the extension of membrane installation lifetimes.

## Figures and Tables

**Table 1:** Characteristics of the membranes used in the present study.

Membrane	Manufacturer	Polymer	MWCO [kDa]	pH Range
FP100	PCI	PVDF	100	1.5–12.0
UE50	TriSep	PES	100	2.0–12.0

**Table 2:** The concentration of major ions in the tap water and NF permeate [mg/L].

Ion	Na^+^	K^+^	Ca^2+^	Mg^2+^	NO_3_^−^	Cl^−^	SO_4_^2−^
Tap water	23.9	5.80	63.90	16.60	1.80	46.90	86.90
NF permeate	2.31	0.35	0.54	0.09	0.17	2.04	0.24

**Table 3:** The results of SEM-EDS analysis. FP100 membrane.

Element	C	O	Fe	Na	Mg	Si	P	Zn	Cl	S
Concentration [wt%]	8.40	58.10	22.60	0.98	0.29	2.40	0.80	1.70	1.30	0.68

**Table 4:** The results of SEM-EDS analysis. The UE50 membrane.

Element	C	O	Fe	Na	Mg	Si	P	Zn	Cl	S
Concentration [wt%]	35.30	36.40	0.17	-	-	0.14	-	0.20	-	23.70

## Data Availability

The data presented in this study are available upon request from the corresponding author. The data are not publicly available due to the institutional repository being under construction.
